# Draft Genome Sequence of Aeromonas caviae UFMG-H8, Isolated from Urine from a Healthy Bovine Heifer (Gyr Breed)

**DOI:** 10.1128/MRA.00388-20

**Published:** 2020-05-07

**Authors:** Silvia Giannattasio-Ferraz, Laura Maskeri, André P. Oliveira, Edel F. Barbosa-Stancioli, Catherine Putonti

**Affiliations:** a Departamento de Microbiologia, Instituto de Ciências Biológicas, Universidade Federal Minas Gerais, Belo Horizonte, Minas Gerais, Brazil; b Bioinformatics Program, Loyola University Chicago, Chicago, Illinois, USA; c Empresa de Pesquisa Agropecuária de Minas Gerais (EPAMIG), Uberaba, Minas Gerais, Brazil; d Department of Biology, Loyola University Chicago, Chicago, Illinois, USA; e Department of Computer Science, Loyola University Chicago, Chicago, Illinois, USA; f Department of Microbiology and Immunology, Stritch School of Medicine, Loyola University Chicago, Maywood, Illinois, USA; University of Maryland School of Medicine

## Abstract

Aeromonas caviae is an emerging pathogen in humans, causing intestinal infections. Here, we report Aeromonas caviae strain UFMG-H8, isolated from the urine of a healthy heifer (Gyr breed).

## ANNOUNCEMENT

Aeromonas caviae is one of the 36 species belonging to the *Aeromonas* genus (1). This species has been reported as an emerging pathogen to humans, causing gastroenteritis and liver cirrhosis and having high rates of drug resistance (2). *A. caviae*, among other species from the genus, has been observed in healthy livestock animals, such as bovines, horses, and sheep, reinforcing the potential of an emerging pathogen for humans in contact with contaminated animals (3). Here, we present the draft genome sequence of *A. caviae* strain UFMG-H8, isolated from a healthy heifer belonging to a herd composed of purebred Gyr cattle.

Sample collection took place in May 2019 at the Agricultural Research Company of Minas Gerais State (EPAMIG) in Brazil. This study was approved by the Ethics Committee in Animal Experimentation of the Universidade Federal de Minas Gerais, Brazil (CEUA/UFMG; 40/2019), and all experiments were performed in accordance with relevant guidelines. For sampling, the vulva was washed with distilled water and soap, and then midstream urine was collected using a 50-ml sterile tube. The material was frozen and kept at –20° overnight until we returned to the lab to process the sample (within 48 h). Then, 2-ml aliquots were centrifuged, and the liquid was plated on a lysogeny broth (LB) agar plate that was incubated at 37°C overnight. Next, single colonies were picked and grown in LB medium at 37°C overnight. This process was repeated 3 times in order to obtain pure colonies. A single colony was picked, inoculated in LB liquid medium, and grown overnight under agitation at 37°C. DNA was extracted using the Qiagen DNeasy UltraClean microbial kit (Qiagen, Hilden, Germany) according to the manufacturer’s instructions and quantified using a Qubit fluorometer. The genus and species were determined through sequencing of the 16S rRNA gene sequence. The DNA was then sent to the Microbial Genomic Sequencing Center (MiGS) at the University of Pittsburgh for whole-genome sequencing. There, the DNA was fragmented using an Illumina tagmentation enzyme, and indices were attached using PCR. The genome was sequenced using the NextSeq 550 platform. Sequencing produced 1,446,892 pairs of 150-bp reads. The raw reads were trimmed using Sickle v1.33 (4) and assembled using SPAdes v3.13.0 with the “only-assembler” option for k values of 55, 77, 99, and 127 (5). After assembly, the genome coverage was calculated using BBMap v38.47 (https://sourceforge.net/projects/bbmap/) and annotated using the NCBI Prokaryotic Genome Annotation Pipeline (PGAP) v4.11 (6). The genome sequence was scanned for antibiotic resistance genes using ResFinder v3.2 (7). Default parameters were used for all software unless otherwise listed.

The draft genome of *A. caviae* strain UFMG-H8 is 4,551,882 bp long, assembled into 33 contigs with a GC content of 59.87%. The assembly has an *N*_50_ value of 410,820 bp and an 85× genome coverage. PGAP annotation identified 4,281 genes, 4,099 of which are predicted to be protein-coding genes, and 104 tRNAs. ResFinder identified antibiotic resistance genes for aminoglycosides, beta-lactams, macrolides, sulfonamides, tetracycline, and trimethoprims. Further analysis of this genome can provide us with a better understanding of *A. caviae* as an emerging pathogen in humans and its presence in healthy cattle.

### Data availability.

This whole-genome sequencing (WGS) project has been deposited in GenBank under the accession number JAAVMO000000000. Raw reads were deposited in the SRA under the accession number SRR11455639. This sample is part of BioProject accession number PRJNA615899.
